# Psychopathology of addiction: Can the SCL90-based five-dimensional structure differentiate Heroin Use Disorder from a non-substance-related addictive disorder such as Gambling Disorder?

**DOI:** 10.1186/s12991-018-0173-7

**Published:** 2018-01-16

**Authors:** Angelo G. I. Maremmani, Denise Gazzarrini, Amelia Fiorin, Valeria Cingano, Graziano Bellio, Giulio Perugi, Icro Maremmani

**Affiliations:** 1 Department of Psychiatry, North-Western Tuscany Region Local Health Unit, Versilian Zone, Viareggio, Italy; 2 Association for the Application of Neuroscientific Knowledge To Social Aims (AU-CNS), Pietrasanta, Italy; 3 G. De Lisio Institute of Behavioural Sciences, Pisa, Italy; 4 Drug Addiction Unit, Castelfranco Veneto, Treviso, Italy; 5 Department of Clinical and Experimental Medicine, Section of Psychiatry, University of Pisa, Pisa, Italy; 6 Vincent P. Dole Dual Diagnosis Unit, Department of Specialty Medicine, Santa Chiara University Hospital, University of Pisa, Via Roma, 67, 56100 Pisa, Italy

**Keywords:** Heroin Addiction, Heroin Use Disorder, Gambling Disorder, Specific psychopathology of Substance Use Disorder, Substance Use Disorders, Psychopathological symptoms

## Abstract

**Background:**

In the Gambling Disorder (GD), there is no exogenous drug administration that acts as the central core of the traditional meaning of addiction. A specific psychopathology of Substance Use Disorders has been proposed recently. In a sample of Heroin Use Disorder (HUD) patients entering opioid agonist treatment, it became possible to identify a group of 5 mutually exclusive psychiatric dimensions: Worthlessness-Being trapped (W-BT), Somatic Symptoms (SS), Sensitivity-Psychoticism (SP), Panic Anxiety (PA) and Violence-Suicide (VS). The specificity of these dimensions was suggested by the absence of their correlations with treatment choice, active substance use, psychiatric comorbidity and the principal substance of abuse and by the opportunity, through their use, of fully discriminating HUD from Major Depression patients and, partially, from obese non-psychiatric patients. To further support this specificity in the present study, we tested the feasibility of discriminating HUD patients from those affected by a non-substance-related addictive behaviour, such as GD. In this way, we also investigated the psychopathological peculiarities of GD patients.

**Methods:**

We compared the severity and frequency of each of the five aspects found by us, in 972 (83.5% males; mean age 30.12 ± 6.6) HUD and 110 (50% males; average age 30.12 ± 6.6) GD patients at univariate (*T* test; Chi square) and multivariate (discriminant analysis and logistic regression) level.

**Results:**

HUD patients showed higher general psychopathology indexes than GD patients. The severity of all five psychopathological dimensions was significantly greater in HUD patients. Discriminant analysis revealed that SS and VS severity were able to discriminate between HUD (higher severity) and GD patients (lower severity), whereas PA and SP could not. W-BT severity was negatively correlated with SS and VS; GD patients were distinguished by low scores for SS and VS low scores associated with high ones for W-BT. Psychopathological subtypes characterized by SS and VS symptomatology were better represented in HUD patients, whereas PA symptomatology was more frequent in GD individuals. No differences were observed regarding the W-BT and SP dimensions. At multivariate level, the one prominent characteristic of HUD patients was the presence of SS (OR = 5.43) as a prominent qualification for psychopathological status.

**Conclusions:**

Apart from the lower severity of all psychopathological dimensions, only the lower frequency of SS typology seems to be the prominent factor in GD patients. The SCL90-defined structure of opioid addiction seems to be useful even in non-substance-related addictive disorders, as in the case of GD patients, further supporting the possible existence of a psychopathology specific to addiction.

## Introduction

In the 5th edition of the Diagnostic and Statistical Manual of Mental Disorders (DSM-5), what had previously been referred to as Pathological Gambling (PG) become Gambling Disorder (GD) and was moved from “Impulse Control Disorder” to the chapter entitled “Substance-Related and Addictive Disorders” [1]. In this way, DSM-5 became the first diagnostic system to recognize a behavioural addiction. In GD, there is no exogenous drug administration that acts as the central core of the traditional meaning of addiction, but people compulsively and dysfunctionally engage in behaviours that can be conceptualized within an addiction framework as different expressions of the same underlying syndrome. In a way parallel to addiction to substances, distinctive elements can be found regarding clinical expression (e.g. craving, tolerance, withdrawal symptoms), comorbidity, neurobiological profile, heritability, and treatment [2]. Further similarities are found between GD and substance use disorders (SUD) in the life course, treatment outcome, diagnostic criteria (though there are differences, too) [3], even if scepticism remains strong [4].

Core elements of addiction include: (1) craving, (2) impaired control over behavioural engagement; and (3) continued behavioural engagement despite adverse consequences. Symptoms of PG and GD in DSM-5 have substantial overlap with the same five symptoms appearing in both diagnostic criteria sets (preoccupation, loss of control, psychosocial consequences in various contexts, tolerance, withdrawal) [3]. Individuals with GD experience a euphoric state similar to a drug-induced ‘‘high’’ [5], symptoms of withdrawal (including restlessness, headaches, and irritability) [6] at levels comparable with those of individuals with alcohol use disorder [6]. 91% of 222 GD patients reported “cravings” which were not related to the use of other drugs [7]. Individuals with GD demonstrate changes in heart rate responses to gambling activities [8] and report increasing levels of gaming or bet size over time [6], phenomena resembling tolerance. This latter effect was linked with the aim of increasing chances of winning rather than improving or maintaining excitement levels [6].

In the development and maintenance of addictive behaviour, the central role of the prefrontal cortex and mesolimbic reward system is highlighted by all the existing theories: reward deficiency hypotheses [9], incentive-sensitization theory [10], impaired response inhibition and salience attribution [11]. Neuroimaging research on SUD has demonstrated changes in fronto-striatal circuits at the brain’s structural and functional levels [12, 13]. The literature supports relatively diminished activation of the prefrontal cortex and subcortical regions (particularly the ventral striatum) [14, 15].

Various cognitive domain alterations (cognitive control, decision-making, reward/loss and “near-miss” processing, delay and probabilistic discounting, reversal learning, alternation learning, and risk-taking) have all been found in people suffering from GD. Nevertheless, the severity of these alterations was at a lower level compared with individuals who had SUD [16]. Indeed, GD patients often show high scores on measures of impulsiveness and low ones on self-control [17]. Concerning impulsivity, in a neurocognitive assessment, individuals with GD and SUD have shown high levels on self-reported measures [18–20]. The first group also did worst on the Iowa Gambling Task, a paradigm that assesses risk-reward decision-making [21].

The Hypothalamic–pituitary–adrenal axis is disrupted by chronic exposure to substances; the same effects are seen during engagement in gambling [22].

Studies on the serotoninergic system corroborated similarity between GD and SUD. The levels of platelet monoamine oxidase (MAO), a peripheral marker of serotonin functions, fall in subjects with GD. Similar findings have been observed in individuals with SUD. Indeed, low levels of the serotonin metabolite 5-hydroxy-indole-acetic acid have been found in the cerebrospinal fluids of people with GD and those with alcoholism [23].

Family cohort studies revealed that GD runs in families, and families with GD frequently have histories of SUD [24]. GD’s heritability rates are similar to those for alcohol and opiates [25].

GD, as diagnosed with DSM-5 criteria, shows a prevalence of about 20.4% in SUD patients [26]. High rates of co-occurrence between SUDs and GD are present in both directions [27]: in GD samples, 50% have a lifetime SUD [28]. Prevalence rates are higher in treatment-seeking samples [29]. Individuals with comorbid GD and alcohol tend to have more severe problems [30]. Indeed, a history of having comorbid SUD often hampers efforts to achieve gambling abstinence. A current alcohol use disorder raises the risk of relapse after gambling treatment, and those with no lifetime history of SUD, compared with those who do have lifetime SUD, are 2.6 times more likely to achieve a 3-month period of gambling abstinence [31].

Despite all these similarities, the specific psychopathology of SUD and GD patients does not appear to have been studied, unless the investigation is related to the presence of a psychiatric comorbidity.

96% of individuals with lifetime GD also meet the criteria for at least one other lifetime psychiatric disorder [32]. The literature estimates comorbidity for those with axis I disorders at 74.8% current and 75.5% lifetime. Among the former group, the highest rates are those referring to current mood disorders (23.1%) with a high incidence of depression [33, 34], alcohol use disorders (AUD) (21.2%), anxiety disorders (17.6%) and substance (non-alcohol) use disorders (7.0%) [35]. Among the latter the highest lifetime rates for comorbidity are those recorded in cases of: mood disorders (49–56%), and anxiety disorders (41–60%), while rates for AUD (73%) and DUD (38%) [32] were shown to be particularly prevalent. Personality disorders are more common among GD patients [36], and the prevalence of multiple comorbid disorders was found to be at a high level as well.

A specific psychopathology of Substance Use Disorders (SUD) has been proposed recently, by applying an exploratory factor analysis to the Symptom Checklist-90 (SCL90), in a sample of HUD patients entering agonist opioid treatment. In this way, it became possible to identify a group of 5 mutually exclusive psychopathological/psychiatric dimensions: Worthlessness-Being trapped (W-BT), Somatic Symptoms (SS), Sensitivity-Psychoticism (S-P), Panic Anxiety (PA) and Violence-Suicide (V-S) [37]. Subsequent studies proved that these dimensions are not substantially influenced by a series of confounding factors. They are, in fact, independent of active heroin use, lifetime psychiatric problems, kind of treatment received or particular drug of abuse (heroin, cocaine or alcohol) [38–41]. As investigated by applying the SCL90 in the studies just mentioned, the five psychopathological dimensions so identified were also correlated with the outcome of a variety of agonist opioid treatments and residential treatments. S-P HUD patients showed better retention in treatment if treated with methadone maintenance, and V-S ones if treated with buprenorphine. V-S HUD patients showed the worst results during a residential treatment [42, 43]. Besides, these five psychopathological dimensions have demonstrated their capability to discriminate HUD patients from other psychiatric patients, specifically those affected by Major Depression [44] and by obesity [45].

Aims: In the present study, we tested the feasibility of discriminating HUD, by applying the 5-dimensional psychopathology, from a non-substance-related addictive behaviour, such as GD. To do that, we have compared HUD with GD patients. Because of the similarities discussed above between SUD and GD, we expected to find no substantial differentiation even in the domain of psychopathology. The secondary aim was to investigate psychopathological peculiarities of GD patients.

## Methods

### Study design and population

Information on patients included in the present study comes from two different datasets. Data regarding HUD patients came from a database including anonymous individual information originally collected for clinical or other research purposes (‘Pisa Addiction Dataset’) at the Dual Diagnosis Unit, Santa Chiara University Hospital in Pisa, Italy. Data regarding GD individuals were extracted from a clinical dataset of patients treated at the Drug Addiction Unit in Castelfranco Veneto, Treviso, Italy. We did not use specific criteria for including patients in these databases other than ‘wish to be treated’ and ‘wanting to participate’ in future survey. The patient could decide independently to accept or decline to be inserted in the database. Acceptance, or a decline, did not affect in any way the care the patient received. The patient could withdraw his/her consent at any time without giving any explanation.

To fulfil the aims of the present analysis, only baseline data were used, implementing a cross-sectional approach. Moreover, specific inclusion criteria were applied: at least 18 years old, with a diagnosis of Heroin dependence, HUD or Pathological Gambling/GD based on DSM-IV/DSM-5 diagnostic criteria, and information available from the SCL-90 questionnaire. Patients undergoing a pharmacological, psychiatric or psychological treatment at baseline were excluded.

The information obtained was analysed after implementing a retrospective, naturalistic, cross-sectional comparative design, with a single evaluation of the patients, and with the purpose of estimating the magnitude of differences, on the severity and typology of psychopathological symptoms, between HUD and GD patients.

All the patients recruited for research aims had signed an informed consent document; patients evaluated for clinical purposes gave their informed consent to the anonymous use of their clinical data for independent studies. Apart from the research protocol, the patients’ assessment was entirely non-interventional, so the choice of the subsequent treatment was made following the conventional criteria that are routinely adopted in clinical practice.

The experimental procedures were approved by the pertinent ethics committees in accordance with internationally accepted criteria for ethical research.

Reflecting inclusion/exclusion criteria, the sample consisted of 1082 patients. Of all these patients, 885 (81.8%) were males, and 197 (18.2%) were females. Mean age was 31.99 ± 9.2 (range 16–74). 972 patients were diagnosed as HUD patients. Of these, 812 (83.5%) were males and 160 (16.5%) females. Mean age was 30.12 ± 6.6 (range 16–59). 110 patients were diagnosed as GD individuals. 53 (50.0%) were males. Mean age was 48.54 ± 12.5 (range 23–74).

### Instruments

#### Symptom Checklist-90 (SCL90)

Developed by Derogatis and colleagues [46], the SCL90 comprises 90 items, on a 5-point scale of distress. It is a self-report clinical rating scale for evaluating current symptomatology of psychiatric outpatients. In the case of substance use disorders, the 90 items reflect five primary symptom dimensions underlying the vast majority of symptomatology that is observed in this class of patients. The primary symptom dimensions are Worthlessness-Being trapped (W-BT), Somatic Symptoms (SS), Sensitivity-Psychoticism (S-P), Panic Anxiety (PA), and Violence-Suicide (V-S) [37].

These five dimensions have been empirically established and primarily validated on a sample involving over 2500 substance use disorder patients [40, 41, 47]. On the basis of the highest z scores obtained on the five SCL90 dimensions, subjects can be assigned to one of five mutually exclusive groups.

The three global scores usually calculated from the SCL90 items are the sum of all elements (Total SCL90); the PST (number of items rated positively), and the PSDI (positive symptom distress index). This latter is calculated by dividing the sum of all elements by the PST.

For a complete description of the SCL90 five-dimensional structure, please see [37, 45].

#### Psychiatric diagnostic evaluation. Structured clinical interview for DSM-IV Axis I disorders, Clinician Version (SCID-I)

This instrument [48] helps clinicians to make standardized, reliable, and accurate diagnoses, and avoid the common problem of ‘premature closure’ (first focusing on one diagnostic possibility). Specially adapted from the research standard for Axis I structured clinical interviewing for use in clinical settings, the SCID-I covers the DSM-IV diagnoses most commonly seen by clinicians. It includes the diagnostic criteria for these disorders with corresponding interview questions. The SCID-I comprises six self-contained modules: mood episodes; psychotic symptoms; psychotic disorders; mood disorders; substance use disorders; and anxiety, adjustment, and other disorders.

### Data analysis

In this study, HUD and GD individuals were compared with demographic and psychopathological dimensions using the Chi-squared test, with Bonferroni’s correction, for categorical variables, and Student’s *t* test for continuous variables.

Differences in the severity of each psychopathological dimension were analysed through the *T* test, at the univariate level, and, using discriminant analysis, at the multivariate level. Differences in typology were assessed by applying the Chi-squared test, at the univariate level, and by using a logistic forward stepwise regression analysis, at the multivariate level, to take into account possible confounding factors. We considered, as criterion, the presence of an HUD diagnosis, and membership of one of the five psychopathological groups, along with various between-group demographic data and degrees of symptomatological severity, as predictors. Statistical routines of SPSS (v20.0) were used.

## Results

### Sociodemographic characteristics and psychiatric-SUD comorbidity

GD patients were significantly (*T* = − 24.72; *p* ≤ 0.001) older (48.54 ± 12.5 years) than HUD ones (30.12 ± 6.5 years). Males were significantly (*χ*^2^ = 19.57; *p* < 0.001) better represented in HUD patients (83.5%) than in GD ones (66.4%). Consequently, females were better represented in GD patients (33.6%) than in HUD ones (16.5%).

GD patients more frequently (*χ*^2^ = 52.68; *p* < 0.001) reached a higher ≥ 8-year educational level than HUD ones (39.1% vs 12.7%, respectively). 44.8% of HUD and 15.5% of GD patients were unemployed (*χ*^2^ = 34.87; *p* < 0.001). No differences were found regarding marital status (*χ*^2^ = 2.07 *p* = 0.173).

13 GD patients showed SUD comorbidity. More specifically, 4 (3.6%) subjects were diagnosed with alcohol use disorder; 3 (2.7%) with stimulant use disorder; 8 (7.3%) with cannabinoid use disorder. None were affected by opioid use disorder, or hallucinogen use disorder or inhalant use disorder. Tobacco use was not considered. Interestingly, GD with and without SUD showed no significant differences when they were compared, in a multivariate way (discriminant analysis), regarding the severity of the five psychopathological dimensions (Wilks’s Lambda = 0.98; df (5); *p* = 0.119). Similarly, no differences emerged when comparing the psychopathological typology (*χ*^2^ = 3.01; df(4); *p* = 0.556).

29 (26.4%) GD subjects were diagnosed as dual disorder (DD) patients. GD with and without DD showed significant difference at multivariate level (Wilks’s Lambda = 0.87; df (5); *p* = 0.014). DD-GD patients showed high worthlessness-being trapped and Sensitivity-Psychoticism symptomatology combined with low Violence-Suicide symptoms. No differences emerged when comparing the psychopathological typology (*χ*^2^ = 2.40; df(4); *p* = 0.662).

### Psychopathological typology in HUD and GD patients

SS was the most represented psychopathological typology in HUD patients. They were characterized by distress arising from perceptions of bodily dysfunction. Patients at treatment entry are characterized by soreness in their muscles, heavy feelings in their arms or legs, having hot and cold spells, nausea or upset stomach, trouble falling sleep, feeling weak in parts of their body, with pain in lower back, feeling low in energy or slowed down. Their sleep is restless or disturbed, and they wake up early in the morning. They report numbness or tingling in parts of their body, a lump in their throat, trembling, heart pounding or racing, trouble getting their breath, poor appetite, pains in heart or chest, feelings of being easily annoyed and irritated. In contrast, GD patients were mostly characterized at treatment entry by a set of symptoms and experiences usually associated clinically with high manifest anxiety. GD patients reported the feeling of being afraid in open spaces or on the streets; they were: afraid to go out of their house alone, afraid to travel on buses, subways, or on trains, afraid their will faint in public. They experienced spells of terror or panic, faintness or dizziness, feelings of fearful. They feel suddenly scared for no reason and had to avoid certain things, places, or activities because they frighten them.

V-S is the least frequent typology of GD patients. This dimension is characterized by three categories of hostile behaviour: thoughts, feelings, and actions, and it comprises thoughts of death and suicidal ideation.

W-BT was the dimension least frequent in HUD but the second best represented in GD patients. This dimension reflects a broad range of the concomitants of the clinical depressive symptoms maximally represented by feelings of worthlessness and of being trapped or caught. At treatment entry, GD patients felt lonely, blue, hopeless about the future, with no interest in things, lonely even when with other people, inferior to others, blocked in getting things done, tense or keyed up, nervous when left alone. They presented trouble in concentrating and had the idea that something was wrong with their mind. They showed loss of sexual interest or pleasure, thinking their mind was going blank, blaming themselves for things. They reported unwanted thoughts, words, or ideas that did not leave their mind. This dimension does not include being troubled by recurrent thoughts of death or suicidal ideation.

### Differences between HUD and GD patients

Table 1 reports differences in psychopathological severity both at univariate and multivariate level. HUD patients showed higher total SCL90 scores, global severity index, number of items rated positively and positive symptom distress index than GD patients. The severity of all the psychopathological dimensions was significantly higher in HUD patients. Discriminant analysis showed that ‘Somatic Symptoms’ and ‘Violence-Suicide’ severity were able to discriminate between HUD (greater severity) and GD patients (less severity), whereas ‘Panic Anxiety’ and ‘Sensitivity Psychoticism’ could not. The ‘Worthlessness-Being trapped’ dimension was negatively correlated with the ‘Somatic Symptoms’ and ‘Violence-Suicide’ dimensions, so that HUD patients were distinguished by high scores for ‘Somatic Symptoms’ and ‘Violence-Suicide’ and by low scores for ‘Worthlessness-Being trapped’. At the reclassification level, 61.0% of HUD patients were confirmed, while 21.8% of GD patients were reclassified as HUD patients. In summary, the severity of psychopathology as a significant parameter allows the differentiation of HUD patients from GD ones.

**Table 1:** Psychopathological severity in HUD and GD patients

	HUD patients *N* = 972	GD patients *N* = 110	*t*	*p*	*DF*
	M ± sd	M ± sd			
Total SCL-90 severity	89.85 ± 57.3	52.77 ± 39.2	7.03	< 0.001	
Global Severity Index	1.00 ± 00.6	0.59 ± 00.4	7.08	< 0.001	
PST, number of	48.79 ± 18.5	31.43 ± 17.4	9.84	< 0.001	
PSDI severity	1.73 ± 00.5	1.54 ± 00.4	3.87	< 0.001	
Psychopathological dimensions					
1-Worthlessness-Being trapped	1.22 ± 0.8	0.83 ± 0.7	5.69	< 0.001	− 0.45
2-Somatic Symptoms	1.29 ± 0.8	0.64 ± 0.5	8.55	< 0.001	0.93
3-Sensitivity-Psychoticism	0.83 ± 0.6	0.51 ± 0.4	5.08	< 0.001	
4-Panic Anxiety	0.46 ± 0.6	0.21 ± 0.3	4.42	< 0.001	
5-Violence-Suicide	0.98 ± 0.7	0.46 ± 0.4	7.12	< 0.001	0.51
*Centroids*	*0.09*	− *0.82*			

*HUD* Heroin Use Disorder, *GD* Gambling Disorder, *PST* positively rated items (number of), *PSDI* positive symptom distress index, *DF* Discriminant function

Statistics: Lambda = 0.92; Chi square = 80.49; df = 5; *p* = < 0.001; 62.8% of original grouped cases correctly classified

Psychopathological subtypes distinguished by “Worthlessness-Being trapped” and “Sensitivity-Psychoticism” symptomatology were unable to differentiate HUD from GD patients. “Somatic Symptoms” and “Violence-Suicide” subtypes were more recurrent in HUD patients, whereas the ‘Panic Anxiety’ subtype was more frequent in GD patients (Table 2).

**Table 2:** Psychopathological typology in HUD patients and PG patients

	HUD patients *N* = 972	GD patients *N* = 110	T/chi	*p*
Psychopathological subtypes				
1-Worthlessness-Being trapped	146 (15.0)a	022 (20.0)a		
2-Somatic Symptoms	248 (25.5)a	10 (09.1)b		
3-Sensitivity-Psychoticism	185 (19.0)a	21 (19.1)a		
4-Panic Anxiety	223 (22.9)a	50 (45.5)b		
5-Violence-Suicide	170 (17.5)a	07 (06.4)b	40.03	< 0.001

At multivariate level, after checking the analysis for age, gender, educational attainment, working activity and severity of psychopathology, the prominent psychopathological characteristic of HUD patients remained their membership of the ‘Somatic Symptoms (OR = 5.43) psychopathological subtype (Table 3). Typology of psychopathology was not the most important factor in discriminating HUD from GD patients (entering step 4 out of 5 in the logistic regression analysis). The “Somatic Symptoms” typology was sufficient to differentiate between the HUD and GD groups.

**Table 3:** Prominent psychopathological characteristics of HUD patients who had been checked for their age, gender, educational level, working activity and severity of symptomatology (only significant results are reported in the present table)

Step		Odds ratio	95% CI	*p*
1	Age	0.78	0.75–0.82	< 0.001
2	Education, low	12.19	5.66–26.2	< 0.001
3	SCL90 total score	1.02	1.01–1.03	< 0.001
4	Psychopathological typology			0.007
	1-Worthlessness-Being trapped	1.00		
	2-Somatic Symptoms	5.43	1.48–19.8	0.010
5	Unemployed	3.07	1.39–6.78	0.005

Statistics: Chi squared 444.18, df 8, *p* < 001, 95.7% of original grouped cases correctly classified

## Discussion

The present study aimed to test whether the specific psychopathology already found in HUD patients could be likewise detected in GD patients. In general, we found that psychopathological symptoms were more severe in HUD patients than in GD ones; more specifically, HUD patients were distinguished by more severe ‘Somatic Symptoms’ and ‘Violence-Suicide’ symptomatology associated with less severe ‘Worthlessness-Being Trapped’ symptoms. ‘Somatic Symptoms’ is the most discriminant psychopathological typology, as it alone is sufficient to differentiate HUD from GD patients at treatment entry.

In one of the first studies on the psychopathological symptoms of gambling disorder, SCL90 items had been compared with PG, psychiatric outpatients and healthy controls, showing significantly lower Obsessive–Compulsive, Interpersonal Sensitivity, Anxiety, and Phobic Anxiety factor scores, but comparable scores on four scales: Somatization, Hostility, Paranoid Ideation, and Psychoticism. Consistently with clinical observations and empirical data reported in the literature, PG patients characteristically showed both cognitive and somatic expressions of clinical depression, including feelings of hopelessness, lack of motivation, suicidal thoughts, and loneliness. The depressive dimension was the only clinical scale to significantly exceed the psychiatric outpatient population sample [49]. GD patients with concurrent SUD showed higher scores on all subscales of the SCL90 than GD patients without concomitant SUD [50]. Three symptomatological clusters were observed by Gonzalez-Ibanez: the highest scores were found on scales of Obsessive–Compulsive, Interpersonal Sensitivity, Depression, and Paranoid Ideation [51]. When dividing GD patients according to age into three groups (17–26 years; 27–43 years and ≥ 44 years), the two older groups showed higher scores, while the youngest stayed in the standard range [52]. Lastly, by comparing single subgroups according to the patients’ age at the onset of GD in each, a higher age at onset of gambling problems proved to be associated with more severe psychopathological symptoms (Depression, Paranoid Ideation and Psychoticism). Conversely, in a study giving results in disagreement with ours, a lower age of onset was related to the greater severity of PG [53]. Cross-sectional and quantitative studies, therefore, showed that high scores for depression were a typical feature of GD patients. In our study, the importance of the depressive dimension (Worthlessness-Being Trapped), in distinguishing GD patients, seems to be confirmed. Further, the depressive symptomatology shows a very similar frequency to that of HUD patients.

The ‘Worthlessness-Being-Trapped’ dimension is the primary factor found in HUD patients [37]. It brings together depressive, obsessive–compulsive and psychotic symptoms, and is dominated by the feeling of uselessness and of being trapped in a corner. This dimension is strictly correlated with the age of patients. Subjects typified by ‘Worthlessness-Being Trapped’ features are, in general, older and, in many cases, have a long history of addictive behaviour [47]. This dimension was unable to significantly differentiate between the pattern of features typifying HUD patients on their entry into Agonist Opioid (AO) or Therapeutic Community (TC) treatment [41]. There was a close similarity, in frequencies, between HUD patients entering TC treatment after detoxification and other patients entering without it [40]. The same thing happened in HUD patients with and without lifetime psychiatric problems [39], and patients using, as their primary substance of use, heroin, cocaine or alcohol [38]. It is probable that this dimension has the status of the most significant psychopathological trait-state of SUD patients. Its special role is further confirmed in the separate case of a non-pharmacological addictive behaviour such as GD. The similarities to be found on neurobiological, genetic and clinical grounds between SUD and GD seem to be confirmed once again at the psychopathological level. Interestingly, the highest scores in this dimension are able to differentiate GD from HUD patients when they are associated with a low degree of severity of the ‘Somatic Symptoms’ and ‘Violence-Suicide’ dimensions. In other words, HUD patients are distinguished by a more severe withdrawal syndrome and aggressiveness, and GD patients by a higher degree of severity of the ‘Worthlessness-Being Trapped’ dimension.

It is, however, possible that the conspicuous presence of aggressiveness in HUD patients was biased by the strong prevalence of male sex in those patients. Male sex is, in fact, already known to correlate with a higher incidence of aggressiveness. Moreover, the multivariate analysis limits the importance of this dimension in discriminating HUD from GD patients.

The ‘Somatic Symptoms’ dimension is distinguished by some physical and anxious elements, which are usually a feature of opiate withdrawal. For this reason, this aspect is more prominent in HUD patients at the start of treatment with AO [41], in non-detoxified HUD patients at TC treatment entry [40], and again, in HUD patients, when they are compared with patients who have cocaine use disorder and/or alcohol use disorder [38]. Nevertheless, the ‘Somatic Symptoms’ dimension was unable to significantly differentiate between HUD patients with and without lifetime psychiatric problems [39]. At this point, it may be stated that this dimension, in general, is more closely related to HUD than to SUD. So the one available logical conclusion seems to be that ‘Somatic Symptoms’ is the only psychopathological aspect capable of differentiating HUD from GD patients.

In summary, in our study, at treatment entry psychopathology was more severe in HUD patients, but its typology was very similar, in any case. This fact is not surprising when considering that HUD and GD often coexist. GD among HUD patients, during methadone maintenance treatment, seems to be a frequent diagnosis (range 7–29%) among the different studies [54–60]. An even higher percentage (52.7%) has been found in patients receiving methadone maintenance treatment; one important finding was that a majority of patients had gambled during the previous 2 years [60].

In our GD sample, we observed the presence of psychiatric and SUD comorbidity, but the severity and typology of psychopathology both proved to be independent of SUD. The severity of symptomatology was linked to the GD, in this study, and, separately, in HUD patients in previous studies, but typology of psychopathology proved to be independent of psychiatric comorbidity in this and in previous studies [38, 39].

### Limitations

The obvious limitations of this study arise from the fact that this is a retrospective analysis carried out on a selected cohort of patients, rather than a study specifically designed to elucidate this issue. Besides, no data were collected about the prevalence of possible Axis II disorders in this sample, and the considerable differences in sample size and composition may have had an adverse impact on the power of the statistical tests that were applied. The strength of the study lies, however, in the care taken in carefully investigating psychiatric comorbidity and SUD in patients with GD, while avoiding the issue that the similarities observed depend on the areas of overlap between the characteristics of the two samples.

## Conclusions

Using the SCL90-defined structure of opioid addiction, we expected to find no substantial differentiation between HUD and GD patients. The possibility of differentiating between groups, using only the SS typology, suggests the applicability of these 5 dimensions even to a non-substance-related addictive disorders, in particular to GD patients, bringing further support to the hypothesis that there is a psychopathology that is specific to addiction.
